# Apixaban- and Rivaroxaban-Associated Bleeding: A Retrospective Analysis Using the FDA Adverse Event Reporting System

**DOI:** 10.7759/cureus.85509

**Published:** 2025-06-07

**Authors:** Ilana K Logvinsky, Christian Sanchez, Claudiu Ciuciureanu, Eric Wu, Osama El Aryan, Maja Delibasic

**Affiliations:** 1 Internal Medicine, Florida State University College of Medicine, Cape Coral, USA; 2 Internal Medicine, Nova Southeastern University, Miami, USA

**Keywords:** anticoagulation, apixaban, bleeding, faers, rivaroxaban

## Abstract

Apixaban and rivaroxaban are two agents commonly used for anticoagulation to treat and prevent blood clots and strokes in individuals with atrial fibrillation (AF). We extracted data from the FDA Adverse Event Reporting System (FAERS) database to evaluate the reporting frequency of ocular, cerebral, gastrointestinal (GI), rectal, and renal hemorrhage, epistaxis, and hemoptysis related to apixaban and rivaroxaban from January 2012 to March 2024. We calculated relative odds ratios (RORs) with 95% confidence intervals (CIs) to compare the hemorrhagic risk profiles of the two agents. There was no statistical significance in reported cases of ocular hemorrhage in apixaban versus rivaroxaban. Based on RORs, rivaroxaban overall has a significantly higher risk of bleeding in multiple organ systems - especially cerebral, GI, rectal, and renal hemorrhage, as well as epistaxis and hemoptysis. Apixaban may be a safer option for individuals at risk for, or with a history of, these complications.

## Introduction

Thromboembolic conditions such as atrial fibrillation (AF) and venous thromboembolism (VTE) require long-term anticoagulation to reduce the risk of stroke and embolism. Direct oral anticoagulants (DOACs) have largely replaced warfarin due to their fixed dosing and predictable pharmacokinetics [1]. Among the DOACs, apixaban and rivaroxaban are two of the most prescribed agents globally [2]. While both drugs are effective, bleeding remains the most clinically significant and potentially life-threatening adverse event (AE) associated with their use [3]. Several randomized trials and real-world observational studies suggest that apixaban may have a more favorable bleeding profile compared to rivaroxaban, particularly regarding major gastrointestinal (GI) and intracranial hemorrhages (ICHs) [4-6]. However, real-world data may complement trial data. Randomized controlled trials, however, have limitations, such as small sample sizes and shorter time frames, which may not be sufficient for detecting rare events [7].

To address some of these limitations, post-marketing surveillance systems, like the FDA Adverse Event Reporting System (FAERS), play an important role in monitoring the safety of medications across diverse patient populations [8,9]. FAERS collects adverse drug events - all reported voluntarily - from healthcare professionals, patients, and even manufacturers, making it a powerful database for the detection of rare but serious AEs, such as cerebral, GI, renal, and other site-specific hemorrhages [10]. Despite the widespread use of both apixaban and rivaroxaban, few pharmacovigilance studies have performed direct comparative analyses of their hemorrhagic safety profiles using FAERS data. The objective of this study is to conduct a retrospective analysis using FAERS data from January 2012 to March 2024 to compare the bleeding-related AE profiles of apixaban and rivaroxaban across multiple organ systems. By quantifying and contrasting the relative odds ratios (RORs) for specific hemorrhagic outcomes, this study aims to provide a broader understanding of the real-world bleeding risks associated with these medications, highlighting potential implications for more individualized prescribing and risk mitigation strategies.

## Materials and methods

Data source and extraction criteria

We analyzed the bleeding risks associated with apixaban and rivaroxaban, specifically focusing on ocular, cerebral, GI, rectal, and renal hemorrhages, as well as epistaxis and hemoptysis. The FAERS is a database that collects reports of AEs, medication errors, and product quality issues resulting in AEs, all submitted to the FDA. The database was accessed via the FAERS public dashboard, with results limited to the period between January 2012 and March 2024. Additionally, the data were filtered to include only cases involving a single offending agent, excluding cases with multiple agents. Duplicate cases were identified by matching case identification numbers and were manually reviewed before being excluded. Each AE was analyzed individually to assess the number of reports.

In the "Listing of Cases," apixaban and rivaroxaban were assessed separately under the suspected product active ingredient. For the "Reaction Group" section, GI hemorrhage was analyzed by grouping several related conditions, such as upper GI hemorrhage, gastric hemorrhage, intra-abdominal hemorrhage, small intestinal hemorrhage, esophageal hemorrhage, gastroduodenal hemorrhage, and intestinal hemorrhage. Hematemesis and rectal hemorrhage were evaluated independently. Renal hemorrhage was listed under the "Renal and Urinary Disorders" section. To assess cerebral hemorrhage, the subcategories of cerebral and ICH were combined under the category of nervous system disorders. Ocular hemorrhage was analyzed by merging the eye and retinal hemorrhage categories under eye disorders. Epistaxis was evaluated under the category of respiratory, thoracic, and mediastinal disorders. This study utilized publicly available datasets; therefore, ethical approval was not required.

Data analysis

To calculate the statistical relationship between apixaban and rivaroxaban based on the listed AEs, we used the odds ratio (OR) and confidence interval (CI). The ROR compares the odds of an event between two groups. The CI provides a range of possible values for the true OR, with the 95% CI being the most common. If the CI includes 1, the OR is considered statistically insignificant, meaning there is no strong evidence to suggest a meaningful difference between the groups. Conversely, if the CI does not include 1, it indicates a statistically significant difference between the groups, suggesting that a real effect is likely present. 

To assess the statistical relationship between apixaban and rivaroxaban based on the reported AEs, the ROR is calculated using a 2 × 2 contingency table for each AE category (such as ocular hemorrhage and cerebral hemorrhage). The table includes data on the number of reported events and non-events for both drugs: the number of events for apixaban (A), the number of non-events for apixaban (B), the number of events for rivaroxaban (C), and the number of non-events for rivaroxaban (D). The non-events are the total AE reports minus the AE in question. The ROR is calculated using the formula: $\text{ROR} = \frac{A \times D}{B \times C}$. This ratio reflects the odds of an AE occurring with one drug compared to the other. An OR of 1 suggests no difference in event likelihood between the two drugs, while a ratio greater than 1 indicates that the numerator drug is more likely to cause the AE, and a ratio less than 1 suggests the denominator drug is more likely. This calculation is repeated for each AE to evaluate the relationship between the two drugs for each specific condition. After obtaining the OR, the natural logarithm (ln) of the ROR is calculated, which makes the calculation of the CI easier. Next, the standard error (SE) of the log-transformed ROR is computed using the formula: $\text{SE}(\ln(\text{ROR})) = \sqrt{\frac{1}{A} + \frac{1}{B} + \frac{1}{C} + \frac{1}{D}}$, where A, B, C, and D are the values from the contingency table. With the SE calculated, you can determine the 95% CI for the log-transformed ROR by applying the formula: $\text{CI} = \ln(\text{ROR}) \pm Z \times \text{SE}(\ln(\text{ROR}))$, where Z is typically 1.96 for a 95% CI [11]. This data was calculated in Excel (Microsoft® Corp., Redmond, WA, USA). Once the CI for the logarithmic ROR is determined, exponentiate the result to return to the original odds ratio scale. The p-value was calculated using Chi-square analysis. This procedure provides the 95% CI for the OR. Calculations were done using Excel software.

## Results

From January 2012 to March 2024, a total of 87,490 AEs were reported for apixaban, while 88,026 AEs were reported for rivaroxaban. Among the reported AEs for apixaban were 200 cases of ocular hemorrhage, 1,097 cases of cerebral hemorrhage, and 1,743 cases of GI bleeding. Additionally, there were 491 cases of rectal bleeding, 58 cases of renal hemorrhage, 1,434 instances of epistaxis, and 278 cases of hemoptysis. For rivaroxaban, the reported hemorrhagic events included 228 cases of ocular hemorrhage, 1,887 cases of cerebral hemorrhage, and 9,440 cases of GI hemorrhage. Furthermore, 2,248 cases of rectal hemorrhage, 223 cases of renal hemorrhage, 2,924 instances of epistaxis, and 889 cases of hemoptysis were documented.

Cerebral hemorrhages were significantly higher for rivaroxaban compared to apixaban, with an ROR of 1.73 (95% CI: 1.60 to 1.86), indicating a higher risk for intracranial bleeding, which can lead to severe morbidity and mortality for patients using this medication. Additionally, GI hemorrhage was also significantly higher in rivaroxaban users, with an ROR of 5.91 (95% CI: 5.61 to 6.22), indicating that GI bleeding is a major concern when prescribing this drug, particularly in elderly patients or those with a history of peptic ulcer disease or GI malignancies.

Rivaroxaban had a significant risk for rectal bleeding, with an ROR of 4.64 (95% CI: 4.21 to 5.12), further supporting its association with GI complications. Rectal bleeding is generally considered less severe and less life-threatening when compared to cerebral hemorrhages; however, it can still pose significant morbidity and may require medical transfusions or surgical interventions if severe. Renal hemorrhages were significantly higher in rivaroxaban users, with an ROR of 3.83 (95% CI: 2.87 to 5.11). This is a concern for patients with chronic kidney disease, as they are already at risk for renal dysfunction and bleeding due to decreased drug clearance. 

Rivaroxaban was associated with a higher risk for epistaxis, with an ROR of 2.06 (95% CI: 1.93 to 2.20), suggesting an increased tendency for mucosal bleeding. Additionally, hemoptysis was a higher risk in patients taking rivaroxaban versus apixaban, with an ROR of 3.20 (95% CI: 2.80 to 3.66), indicating a greater pulmonary bleeding risk. Hemoptysis can be a severe and distressing symptom, particularly in patients with underlying pulmonary conditions such as chronic obstructive pulmonary disease or lung malignancies. There was no statistically significant difference between the two drugs in the occurrence of ocular hemorrhage, suggesting a comparable risk in this specific category. Results are presented in Table 1.

**Table 1:** Results of adverse events (AEs) with relative odds risk (ROR) and 95% confidence interval (CI).

	ROR	95% CI	p-value
Ocular Hemorrhage	1.13	0.94 to 1.37	p = 0.1968
Cerebral Hemorrhage	1.73	1.60 to 1.86	p < 0.0001
Gastrointestinal Hemorrhage	5.91	5.61 to 6.22	p < 0.0001
Rectal Hemorrhage	4.64	4.21 to 5.12	p < 0.0001
Renal Hemorrhage	3.83	2.87 to 5.11	p < 0.0001
Epistaxis	2.06	1.93 to 2.20	p < 0.0001
Hemoptysis	3.20	2.80 to 3.66	p < 0.0001

## Discussion

Retrospective analysis of the FAERS from January 2012 to March 2024 revealed a significantly higher number of hemorrhagic events associated with rivaroxaban, when compared to apixaban. These findings are consistent with the existing literature described below and provide valuable insight into the real-world safety profiles of these anticoagulants [1-5].

In nonvalvular AF, known trials established that apixaban is as effective as warfarin for preventing stroke or embolization, while rivaroxaban is non-inferior. In the ARISTOTLE trial, apixaban was superior to warfarin for stroke prevention (1.27% vs. 1.60% per year) [6]. In the ROCKET-AF trial, rivaroxaban met noninferiority (stroke rates ~2.1% vs. 2.4% per year), but didn’t meet superiority over warfarin [8]. Real-world evidence suggests apixaban’s efficacy is at least comparable - and possibly better - than that of rivaroxaban in practice. A meta-analysis of 10 observational studies in AF found apixaban users had a significantly lower hazard of stroke or systemic embolism than rivaroxaban users (pooled HR (hazard ratio): ~0.88) [9]. Similarly, a large cohort study in routine care reported no loss of efficacy with apixaban: rivaroxaban was not associated with any lower risk of ischemic stroke than apixaban [10]. 

In treating VTE, both agents were found to have the same efficacy in their phase III trials. Apixaban (AMPLIFY trial) was found to be a non-inferior option to conventional enoxaparin-warfarin therapy for acute VTE, with a 2.3% vs. 2.7% incidence of a recurrent VTE event [12]. Rivaroxaban similarly provided non-inferiority to enoxaparin/vitamin K antagonist therapy in the EINSTEIN-DVT and pulmonary embolism trials [13]. To compare more real-life data and uses, in a large U.S. cohort of nearly 37,000 patients per group, new users of apixaban for acute VTE had significantly lower rates of recurrent VTE than those on rivaroxaban (HR: 0.77, 95% CI: 0.69-0.87) [14].

Apixaban and rivaroxaban were both found to have an improved safety profile compared to warfarin, but important differences do exist between them regarding the risk of bleeding as an adverse effect. In the ARISTOTLE trial, apixaban was shown to significantly reduce major bleeding by 31% relative to warfarin (2.13% vs. 3.09% per year) [6], a significant difference. Rivaroxaban’s overall major bleeding rate in the ROCKET-AF trial was found to be statistically similar to warfarin (≈3.6% vs. 3.4% per year) [8], although rivaroxaban did lower the incidence of the most critical bleeding events (intracranial and fatal hemorrhages) when compared to warfarin [8]. A 2020 observational study in AF found major bleeding rates were significantly lower with apixaban (1.8 per 100 patient-years) than with rivaroxaban (2.9 per 100 patient-years) (HR: ~0.64) [9]. Similarly, when looking at real-world data, a meta-analysis including AF data showed a 38% lower risk of major bleeding with apixaban when compared to rivaroxaban (HR: 0.62, 95% CI: 0.56-0.69) [9]. Notably, a nationwide Medicare analysis of elderly AF patients found rivaroxaban was associated with a substantially higher risk of major extracranial bleeding than apixaban (adjusted HR: 2.70) [15].

The difference between apixaban and rivaroxaban is best shown when tracking the events of GI bleeds. Rivaroxaban’s once-daily dosing produces higher peak anticoagulant levels, which may contribute to GI bleeding when compared to the twice-daily dosing schedule of apixaban, which produces a lower and more sustained peak. In the ROCKET-AF trial, major GI bleeding was more commonly seen with rivaroxaban than with warfarin [8], whereas apixaban showed no significant increase in GI hemorrhage versus warfarin in the ARISTOTLE trial [6]. A network meta-analysis confirmed apixaban’s advantage: among standard-dose DOACs, apixaban was associated with the lowest risk of major GI bleeding [16]. Numerous real-world studies echo this finding. In an observational AF meta-analysis, apixaban users had a 43% lower hazard of GI bleeding when compared to rivaroxaban users (HR: ~0.57) [9]. Another large cohort of older patients reported that apixaban was associated with significantly less GI bleeding than rivaroxaban (around 33% lower relative risk) [15].

By contrast, ICH rates are low with both agents and do not differ dramatically. Apixaban and rivaroxaban both markedly lower the risk of ICH relative to warfarin - on the order of a 50%-60% risk reduction in trials [6,8]. Apixaban nearly halved the incidence of intracranial bleeding vs. warfarin in the ARISTOTLE trial (HR: ~0.42) [6], and rivaroxaban similarly reduced ICH vs. warfarin in ROCKET-AF (0.5% vs. 0.7% per year, p = 0.02) [8]. When comparing apixaban to rivaroxaban directly, most analyses find no statistically significant difference in rates of intracranial bleeds [8,15]. Nevertheless, some evidence hints that apixaban might have a slight edge. A recent nationwide cohort study observed a modest but significant reduction in ICH risk with apixaban compared to rivaroxaban (adjusted HR: ~0.85) [1].

It is also important to consider other sites of potential hemorrhages, including genitourinary bleeding. Apixaban may show a lower risk of hematuria or related complications compared to rivaroxaban. When looking at real-world data, we can evaluate one large cohort study where apixaban was associated with significantly fewer urogenital bleeding events than rivaroxaban (approximately 17%-18% relative risk reduction) [1]. Another analysis noted that rivaroxaban users had a higher incidence of hematuria requiring medical attention than apixaban users [1].

A growing body of evidence from randomized trials, meta-analyses, and real-world data indicates that apixaban generally has a more favorable safety profile than rivaroxaban regarding bleeding complications. Apixaban is associated with significantly lower rates of major bleeding, especially when comparing the rate of GI hemorrhage [9,15]. Intracranial bleeding rates are low with both agents, though some data suggest that apixaban may have a slight advantage [1,6]. The American Geriatrics Society, in 2023, recommended avoiding long-term rivaroxaban in older adults when safer alternatives like apixaban are available, due to these bleeding concerns [17].

Our FAERS findings are consistent with this literature and support the notion that apixaban may be a safer choice for patients at elevated bleeding risk, without compromising efficacy in preventing strokes in AF or recurrent VTE events. More studies are needed to compare these two options head-to-head, to favor one over the other, and to help aid in pairing patients with the most appropriate therapy. This study does not take into account the dosages of these medications.

Limitations

The FAERS collects reports on drugs and biologics, but it’s important to remember that inclusion in a report does not prove that the drug or biologic caused the AE. FAERS data should not be relied upon alone to assess a drug’s or biologic’s safety profile. There are several limitations to consider, such as the possibility of duplicate or incomplete reports, since it is a spontaneous reporting system, and causality is not determined from any single report. Another limitation of the FAERS database is the potential for underreporting and a lack of causality assessment. Additionally, patient demographics are not listed in the database. 

The event could be due to the underlying condition, other medications, or other contributing factors. Additionally, the information provided in these reports has not been medically verified and reflects only the observations of the person submitting the report. This dataset cannot be used to estimate the true incidence or prevalence of AEs. As a result, patients should always consult their healthcare provider before making any changes to their medication. Switching therapies should be individualized based on bleeding risk, renal function, and thrombotic risk, as direct comparative switching trials are limited.

## Conclusions

In this comprehensive analysis of FAERS data from January 2012 to March 2024, rivaroxaban was associated with significantly higher RORs of hemorrhagic events compared to apixaban across nearly all examined bleeding sites. Rivaroxaban showed increased signal strength for cerebral, GI, rectal, renal, epistaxis, and hemoptysis events. These findings are consistent with the observed case counts, where rivaroxaban was linked to nearly double, or even more than double, the number of bleeding reports. No significant difference was observed in ocular hemorrhage between the two agents, suggesting a comparable risk at that site. The increased bleeding signals associated with rivaroxaban raise important clinical considerations, especially for patients with a history of GI pathology, chronic kidney disease, pulmonary conditions, or prior intracranial bleeding. These findings can help clinicians guide treatment based on individual patient backgrounds.

These results reinforce prior observational studies suggesting a more favorable bleeding profile for apixaban and highlight the importance of individualized DOAC selection, but they should be interpreted with caution due to the inherent limitations of spontaneous reporting systems. Surveillance databases such as FAERS provide valuable real-world insight into drug safety beyond the scope of clinical trials, though they have limitations. New prospective studies are needed to investigate the underlying mechanisms driving these site-specific bleeding risks.
